# Philadelphia Hospital

**Published:** 1859-01

**Authors:** 


**PHILADELPHIA HOSPITAL.**

*Blockley.*

It is understood that Clinical Lectures will be speedily commenced in the amphitheatre of this immense hospital, and an opportunity given to the student for personal inspection of disease in its extensive wards.

A class has already engaged to attend, and we commend the matter to the attention of students, generally, as a means of acquiring a knowledge of as great a variety of disease as is presented by any institution of the kind in this country.

The suburban location of the building will make a resort to it by the student a pleasant recreation, relieving his pent-up existence, and varying the changeless scene of streets and brick walls.

The distance of the hospital from the centre of the city is but a moderate walk, or is quickly accessible by one of the passenger railways.

Through the energy of Dr. Oliver, a member of the Board of Guardians, all impediments to the permanent establishment of the Clinics have been removed, and great addition to the hospital advantages of this city has been gained.

The Clinics will be continued twice a week throughout the year.

At a recent meeting of the managers of this hospital, the following Consulting Board was appointed, who with the Chief Resident Physician will conduct the Clinics.

*Physicians.*—Drs. J. Carson, S. H. Dickson, J. B. Biddle, J. A. Meigs.

*Surgeons.*—Drs. S. Neill, D. H. Agnew, R. J. Levis, W. S. Halsey.

*Obstetricians.*—Drs. R. A. F. Penrose, E. McClellan.

*Medical & Surgical Reporter.*

